# Advanced HIV Presenting With Pancytopenia and HLH‐Like Hyperinflammation Secondary to Disseminated Talaromycosis: A Case Report

**DOI:** 10.1155/crdi/9257582

**Published:** 2026-07-29

**Authors:** Zhong Xhen Khor

**Affiliations:** ^1^ Department of Internal Medicine, IMU University, Seremban, Negeri Sembilan, Malaysia, imu.edu.my

**Keywords:** haemophagocytic lymphohistiocytosis, HIV, opportunistic infection, pancytopenia, *Talaromyces marneffei*

## Abstract

Haemophagocytic lymphohistiocytosis (HLH) is a life‐threatening hyperinflammatory syndrome that may complicate advanced HIV infection, most commonly triggered by opportunistic infections. We report a man with advanced HIV who presented with prolonged fever and pancytopenia without an identifiable source despite extensive investigation. Bone marrow examination demonstrated haemophagocytosis, while blood mycobacterial culture yielded *Penicillium marneffei*, now classified as *Talaromyces marneffei*. Together with prolonged fever, pancytopenia, marked hyperferritinaemia and a retrospective HScore of 189, these findings supported disseminated talaromycosis with an HLH‐like hyperinflammatory syndrome; complete HLH‐2004 testing was not available. The patient demonstrated rapid clinical and haematological recovery following antifungal therapy with amphotericin B, without the need for HLH‐directed immunosuppressive treatment. Antiretroviral therapy was subsequently initiated, followed by successful treatment of chronic hepatitis C infection. This case highlights the importance of early consideration of fungal infections and HLH‐like syndromes in advanced HIV, as well as the diagnostic value of bone marrow examination in identifying treatable causes of prolonged febrile illness.

**Learning points**
• Persistent fever with pancytopenia in advanced HIV should prompt consideration of disseminated talaromycosis and associated HLH‐like hyperinflammation.
• Blood mycobacterial culture and bone marrow examination may provide complementary diagnostic information, identifying the infectious trigger and demonstrating haemophagocytosis.
• Treatment should prioritise the underlying fungal infection, while adjunctive HLH‐directed immunomodulatory therapy should be considered individually according to disease severity and clinical response.

## 1. Introduction

Haemophagocytic lymphohistiocytosis (HLH) is a severe hyperinflammatory syndrome characterised by uncontrolled immune activation, leading to fever, cytopenias and multiorgan dysfunction. In adults, HLH is most commonly secondary to infections, malignancy or autoimmune disease. In people living with HIV, HLH is more frequently triggered by opportunistic infections rather than by HIV itself [1, 2].

*Talaromyces marneffei* is an endemic opportunistic fungal pathogen in Southeast Asia and a recognised cause of disseminated infection in advanced HIV [3]. Although talaromycosis is well described, its association with HLH remains uncommon, with only a limited number of adult cases reported. Early recognition is critical, as timely treatment of the underlying infection may be lifesaving [3, 4].

## 2. Case Presentation

A man in his 30s with a history of intravenous drug use presented with a 6‐month history of intermittent fever and chronic diarrhoea, without respiratory symptoms.

He had a background of advanced HIV infection, chronic hepatitis C infection and presumed pleural tuberculosis, with poor adherence to both antitubercular and antiretroviral therapy. Review of prior records revealed negative pleural mycobacterial cultures.

On admission, he was febrile and clinically unwell. Examination revealed oral candidiasis, with no focal source of infection or organomegaly. Laboratory investigations demonstrated pancytopenia with marked lymphopenia (absolute lymphocyte count: ∼0.5 × 10^9^/L), elevated liver enzymes and hypoalbuminaemia (Table 1). HIV viral load was markedly elevated (5,883,287 copies/mL) with severe immunosuppression (CD4 count: 30 cells/μL).

**TABLE 1:** Serial laboratory parameters during hospitalisation.

	SI unit	Reference	Day 1	Day 7	Day 14	Day 21	Day 28	6 month
Hb	g/dL	13.0–17.0	5.8	7.9	7.2	7.1	7.6	13.9
WBC	× 10^9^/L	4.0–11.0	1.1	1.9	3.2	6.4	4.0	7.3
Platelet	× 10^9^/L	150–400	52	81	138	220	198	274
HCT	%	40–50	18.7	23.6	22.8	21.3	21.5	40.9
Na	mmol/L	135–145	132	132	142	143	144	145
K	mmol/L	3.5–5.0	3.9	3.5	2.7	3.1	3.0	4.2
Cl	mmol/L	98–107	99	99	108	114	111	109
Creatinine	μmol/L	60–110	85	76	91	83	97	81
Urea	mmol/L	2.5–7.5	2.8	2.4	5.7	6.3	7.3	2.9
Protein	g/L	60–80	—	64.8	60.5	56.5	56.6	78
Albumin	g/L	35–50	16.4	16.3	18.6	22.3	19.6	44.8
Globulin	g/L	20–35	—	—	—	34	37	34
Bilirubin	μmol/L	< 21	22	24.7	14.6	10.3	12.4	10.9
ALP	U/L	30–120		168	173	106	95	122
ALT	U/L	< 40	101	96.8	89	35.7	36.3	45.9
Ferritin	μg/L	30–400	—	—	19,092	—	1926	—
LDH	U/L	120–250	—	—	—	—	219	—
Corrected calcium	mmol/L	2.10–2.60	2.2	2.2	—	2.2	—	—
Magnesium	mmol/L	0.70–1.10	—	—	0.7	0.83	—	—
Phosphate	mmol/L	1.1–1.8	—	—	—	0.8	—	—

*Note:* Day 1 refers to hospital admission. Day 14 refers to the date when bone marrow aspiration and trephine biopsy were performed. Ferritin was measured after bone marrow examination when HLH‐like hyperinflammation was suspected. Day 21 refers to approximately 1 week after initiation of intravenous amphotericin B. The 6‐month column refers to outpatient follow‐up after completion of acute treatment.

An extensive diagnostic workup, including blood cultures, chest radiography, sputum, urine and stool studies, echocardiography and abdominal ultrasonography, was nondiagnostic. Contrast‐enhanced CT imaging demonstrated bowel wall thickening with mesenteric lymphadenopathy, raising suspicion for gastrointestinal tuberculosis; however, subsequent gastric lavage, stool acid‐fast bacilli studies and colonoscopy were unremarkable.

**FIGURE 1:**
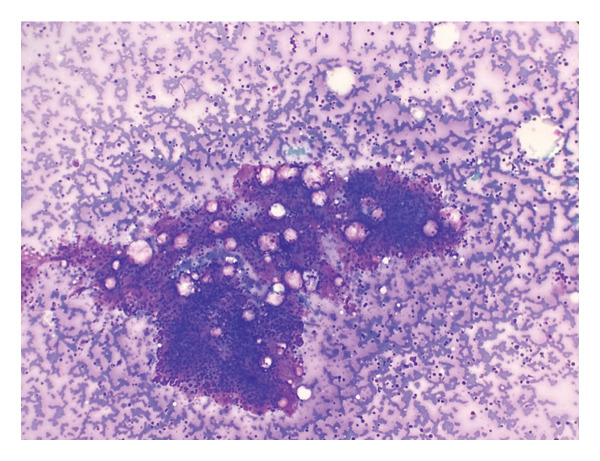
(× 100) Bone marrow aspirate smear (May–Grünwald–Giemsa, 10× objective) showing numerous histiocytes with evidence of haemophagocytosis.

**FIGURE 2:**
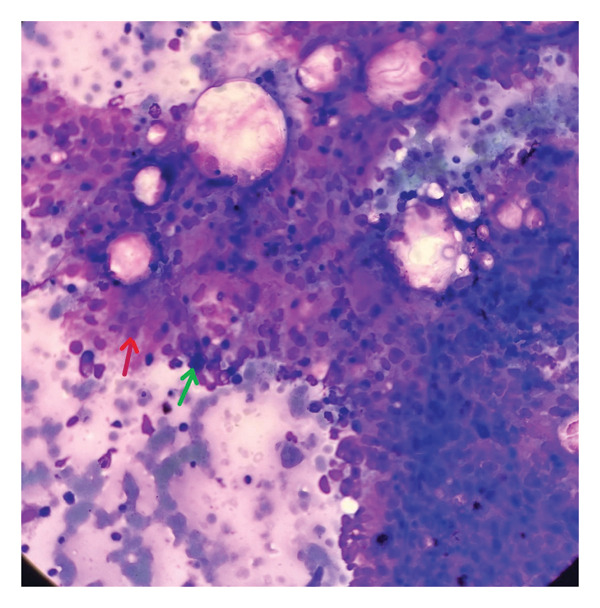
(× 400) Higher magnification showing a histiocyte containing an erythroid precursor (red arrow) and a neutrophil (green arrow), consistent with haemophagocytosis.

**FIGURE 3:**
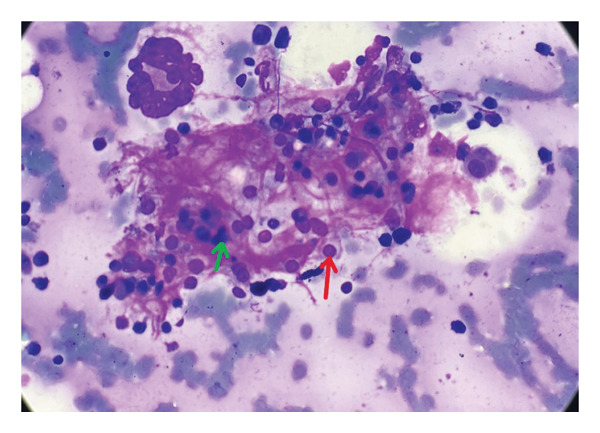
(× 600) High‐power view demonstrating a haemophagocytic histiocyte containing erythroid, neutrophilic and lymphoid elements (green arrow).

Despite 2 weeks of intravenous meropenem and empirical second‐line antitubercular therapy, the patient remained persistently febrile with worsening cytopenias. In view of the nonresolving course, mycobacterial blood culture and bone marrow examination were performed. Blood mycobacterial culture demonstrated fungal hyphae and subsequently yielded *Penicillium marneffei*, now classified as *Talaromyces marneffei*. Bone marrow examination demonstrated haemophagocytosis (Figures 1–3). Serum ferritin, obtained subsequently, was markedly elevated at 19,092 μg/L.

Although this patient exhibited several clinicopathological features suggestive of secondary HLH and had a retrospective HScore of 189, corresponding to an estimated 80%–88% probability of HLH, complete HLH‐2004 testing was unavailable. Triglycerides, fibrinogen, soluble CD25 and natural killer cell activity were not obtained because HLH was not initially suspected. We therefore consider the case best framed as disseminated talaromycosis with an HLH‐like hyperinflammatory syndrome, or at most probable secondary HLH.

The patient was treated with intravenous amphotericin B deoxycholate (0.7 mg/kg daily) for 14 days, followed by oral fluconazole. During amphotericin B therapy, the patient developed hypokalaemia, with a nadir serum potassium of 2.7 mmol/L. This was managed with electrolyte monitoring and potassium replacement, without interruption of antifungal therapy. Antiretroviral therapy (tenofovir disoproxil fumarate, emtricitabine and efavirenz) was initiated after completion of antifungal induction therapy to minimise the risk of immune reconstitution inflammatory syndrome. No HLH‐directed immunosuppressive therapy was administered.

The patient demonstrated rapid clinical and haematological recovery, with resolution of fever and cytopenias. He was discharged with close follow‐up and directly observed therapy. Over subsequent months, he showed sustained improvement, including weight gain, functional recovery and immune reconstitution (CD4 > 200 cells/μL). Empirical antitubercular therapy was completed despite negative cultures, and hepatitis C infection was successfully treated with direct‐acting antivirals. There has been no recurrence of opportunistic infection or HLH on follow‐up.

## 3. Discussion

Disseminated *Talaromyces marneffei* is a recognised opportunistic pathogen in advanced HIV, particularly in endemic regions of Southeast Asia. However, its association with HLH remains uncommon in adults, with only a limited number of cases reported. In this setting, HLH is more frequently triggered by opportunistic infections than by HIV itself, and clinical features such as prolonged fever and cytopenias often overlap with disseminated infection, contributing to diagnostic delay [1–3].

In our patient, the diagnosis was delayed due to a nonspecific presentation and an initially nondiagnostic workup. Bone marrow examination was pursued after persistent fever and progressive cytopenias despite antimicrobial and empirical antitubercular therapy and demonstrated haemophagocytosis. In parallel, blood mycobacterial culture yielded *Penicillium marneffei*, now classified as *Talaromyces marneffei*, confirming disseminated talaromycosis. This combination of microbiologically confirmed opportunistic fungal infection and marrow haemophagocytosis provided the diagnostic basis for an HLH‐like hyperinflammatory syndrome. Similar observations have been reported in prior cases, where bone marrow evaluation facilitated both microbiological diagnosis and recognition of associated hyperinflammatory syndromes [4–10]. Reported adult cases are summarised in Table 2.

**TABLE 2:** Reported adult cases of *Talaromyces marneffei* infection associated with haemophagocytosis, HLH or HLH‐like hyperinflammation.

Author (year)	HIV status	CD4 (cells/μL), if reported	Ferritin (μg/L)	Diagnosis of TM	Treatment of TM	Basis of HLH	HLH‐directed therapy	Outcome
Chim et al. [4]	NR	NA	NR	Culture (site NR)	Amphotericin B	Marrow haemophagocytosis	NR	Survived
Pei [5]	Positive	NR	NR	Blood and marrow	Amphotericin B ⟶ itraconazole	HLH‐2004 criteria	NR	Survived
Pan [6], adult patient 1	Negative	NA	747	Skin	Fluconazole	HLH‐2004	None	Died
Pan [6], adult patient 2	Negative	NA	46,020	Skin, lymph node	Voriconazole	HLH‐2004	Steroids	Unknown
Pan [6], adult patient 3	Negative	NA	8966	Sputum, marrow	Fluconazole ⟶ voriconazole	HLH‐2004	Steroids + IVIG	Survived
Cheok [7]	Positive	NR	NR	Marrow	Antifungal, not specified	Marrow haemophagocytosis	None	Survived
Wu [8]	Positive	16	> 4000	Blood + marrow	Amphotericin B ⟶ itraconazole	HLH‐2004	Steroids	Survived
Hu [9]	Negative	NA	1650	Marrow	Antifungal therapy	Marrow haemophagocytosis	Steroids ± IVIG	Died
Yang [10]	Negative	NA	> 40,000	Blood + marrow	Amphotericin B	HLH‐2004	Steroids	Died
Present case	Positive	30	19,092	Blood *Mycobacterium* cultures	Amphotericin B ⟶ fluconazole	Marrow haemophagocytosis; ferritin: 19,092 μg/L; retrospective HScore: 189; incomplete HLH‐2004 testing	None	Survived

*Note:* IVIG: intravenous immunoglobulin; HLH: haemophagocytic lymphohistiocytosis; HScore: haemophagocytic syndrome diagnostic score. *Penicillium marneffei* is the former nomenclature for *Talaromyces marneffei*.

Abbreviations: NA, not applicable; NR, not reported; TM, *Talaromyces marneffei*.

Although a complete HLH‐2004 diagnostic evaluation was not performed, retrospective clinical assessment suggests a high probability of HLH based on the presence of fever, cytopenias, marked hyperferritinaemia and bone marrow haemophagocytosis. HLH remains a clinicopathological diagnosis supported by a constellation of findings, and incomplete fulfilment of formal criteria is common in real‐world practice [1, 2]. Importantly, haemophagocytosis alone is not diagnostic of HLH, but in the appropriate clinical context, it supports the presence of a hyperinflammatory syndrome. Reported mortality of secondary HLH remains high, particularly when diagnosis and treatment are delayed [2].

Management of infection‐associated HLH in immunocompromised patients remains challenging. HLH‐directed immunosuppressive therapy may be required in severe, progressive or refractory cases, particularly when there is worsening organ dysfunction, severe cytopenia, bleeding risk or poor response to antimicrobial therapy. In previously reported cases of *T. marneffei*–associated HLH or haemophagocytic hyperinflammation, adjunctive immunosuppressive therapy was used in some patients with variable outcomes [4–10]. In our patient, clinical and haematological recovery occurred after antifungal therapy without corticosteroids, etoposide or other HLH‐directed immunosuppression. However, this single case cannot establish antifungal therapy alone as a general treatment strategy, and decisions regarding adjunctive immunomodulatory therapy should be individualised according to severity and clinical response.

This case also highlights the complementary roles of microbiological culture and bone marrow examination. Blood mycobacterial culture established the diagnosis of disseminated talaromycosis, while bone marrow examination demonstrated haemophagocytosis and supported the presence of an associated hyperinflammatory syndrome. In patients with advanced HIV, prolonged fever and unexplained cytopenias, parallel microbiological investigation and bone marrow evaluation may therefore facilitate both pathogen identification and recognition of HLH‐like inflammatory complications [3].

This case has several limitations. First, the case occurred several years before manuscript preparation, and some primary laboratory materials, including the original culture isolate and additional pathology material for fungal staining, were not available for re‐examination. However, contemporaneous microbiology documentation confirmed growth of *Penicillium marneffei* from blood mycobacterial culture. Second, a complete HLH‐2004 diagnostic evaluation was not performed because HLH was not initially suspected; triglycerides, fibrinogen, soluble CD25 and natural killer cell activity were unavailable. Third, as a single case report, this observation cannot determine the optimal role of adjunctive HLH‐directed immunosuppressive therapy in talaromycosis‐associated hyperinflammation.

Finally, this case underscores the importance of careful therapeutic sequencing in patients with multiple coexisting infections. Antifungal therapy was prioritised, followed by delayed initiation of antiretroviral therapy to minimise the risk of immune reconstitution inflammatory syndrome and subsequent treatment of hepatitis C infection. Such a stepwise approach is essential to optimise outcomes while minimising drug–drug interactions and treatment‐related complications in complex immunocompromised patients [11, 12].

## 4. Conclusion

In advanced HIV with prolonged fever, pancytopenia and hyperferritinaemia, disseminated talaromycosis should be considered as a potential infectious trigger of HLH‐like hyperinflammation. Blood mycobacterial culture may establish the microbiological diagnosis, while bone marrow examination can demonstrate haemophagocytosis and support recognition of an associated hyperinflammatory syndrome. Early antifungal therapy is essential, while the need for adjunctive HLH‐directed immunomodulatory therapy should be individualised according to clinical severity and response.

## Author Contributions

Zhong Xhen Khor conceptualised the study, collected the data, drafted the article and revised the manuscript.

## Funding

No funding was received for this work.

## Disclosure

Zhong Xhen Khor is the guarantor of the manuscript and takes responsibility for the integrity of the work as a whole. The author approved the final copy.

## Ethics Statement

Written informed consent for publication of the clinical details and accompanying images was obtained from the patient.

## Conflicts of Interest

The author declares no conflicts of interest.

## Data Availability

Data sharing is not applicable to this article as no datasets were generated or analysed during the current study.
